# Optimal therapeutic conditions for the neural stem cell-based management of ischemic stroke: a systematic review and network meta-analysis based on animal studies

**DOI:** 10.1186/s12883-022-02875-z

**Published:** 2022-09-13

**Authors:** Yongna Yang, Xurui Hu, Qijie Qin, Fanling Kong, Xiaolan Peng, Jing Zhao, Jianghua Si, Zhilong Yang, Shoupin Xie

**Affiliations:** The first people’ s hospital of lanzhou city, Lanzhou, 730000 China

**Keywords:** Ischemic stroke, Neural stem cells, Therapeutic strategies, Animal studies, Systematic review, Network meta-analysis

## Abstract

**Background:**

In order to promote the clinical translation of preclinical findings, it is imperative to identify the most optimal therapeutic conditions and adopt them for further animal and human studies. This study aimed to fully

explore the optimal conditions for neural stem cell (NSC)-based ischemic stroke treatment based on animal studies.

**Methods:**

The PubMed, Ovid-Embase, and Web of Science databases were searched in December 2021. The screening of search results, extraction of relevant data, and evaluation of study quality were performed independently by two reviewers.

**Results:**

In total, 52 studies were included for data analysis. Traditional meta-analysis showed that NSCs significantly reduced the modified neurological severity score (mNSS) and volume of cerebral infarct in animal models of ischemic stroke. Network meta-analysis showed that allogeneic embryonic tissue was the best source of NSCs. Further, intracerebral transplantation was the most optimal route of NSC transplantation, and the acute phase was the most suitable stage for intervention. The optimal number of NSCs for transplantation was 1–5×10^5^ in mouse models and 1×10^6^ or 1.8×10^6^ in rat models.

**Conclusions:**

We systematically explored the therapeutic strategy of NSCs in ischemic stroke, but additional research is required to develop optimal therapeutic strategies based on NSCs. Moreover, it is necessary to further improve and standardize the design, implementation, measuring standards, and reporting of animal-based studies to promote the development of better animal experiments and clinical research.

**Supplementary Information:**

The online version contains supplementary material available at 10.1186/s12883-022-02875-z.

## Background

Stroke is the second leading cause of death globally, accounting for approximately 11.6% of the overall global mortality, i.e., more than 6.55 million deaths each year. The Global Burden of Disease Study 2019 reported 12.2 million new cases of stroke and a stroke prevalence of 101 million cases worldwide, 62.4% of which involved ischemic stroke[1]. Currently, the rapid administration of intravenous recombinant tissue-type plasminogen activator (r-tPA) is the most effective treatment for ischemic stroke[2]. However, around 90% of patients cannot fully benefit from r-tPA because of its short therapeutic window (3–4.5 h post-stroke) and risk of severe complications such as intracranial hemorrhage. Moreover, even the early administration of r-tPA cannot prevent neuronal death in the brain [3–6]. Although endovascular techniques such as endovascular mechanical thrombectomy can extend the therapeutic window to some extent (6–24 h), owing to their strict indications (e.g., time of onset, lesion location, and extent of vascular obstruction), these techniques are not suitable for several patients [7]. Currently, only 13–20% of patients with acute ischemic stroke are potentially eligible for mechanical thrombectomy [8, 9]. Furthermore, this procedure has not been tested in high-quality randomized controlled trials, hindering its widespread adoption in clinical practice [10]. Therefore, ischemic stroke represents a serious public health problem worldwide, and safer and more effective alternative therapies are urgently required.

Recently, progress in regenerative medicine and deeper insights into the pathophysiology of ischemic stroke have generated new hope for its treatment. Stem cell therapy is considered to be the most promising therapeutic strategy for ischemic stroke and can greatly extend the therapeutic window [11]. Given the intensive research on the mechanisms involved in stem cell-mediated repair and continuous advances in isolation, culture, induction, monitoring, and transplantation techniques, stem cells have become a hotspot for the treatment of ischemic stroke [12, 13]. In particular, neural stem cells (NSCs) can promote the recovery of neurological function in stroke patients by protecting the blood–brain barrier, reducing the inflammatory response, and promoting neurogenesis and angiogenesis. In addition, there are abundant sources of NSCs, as NSCs can be directly obtained from neural tissue or generated from the differentiation of embryonic stem cells or induced pluripotent stem cells. Moreover, NSCs have a strong potential for neuronal phenotypic differentiation, chemotaxis, and migration *in vivo* and *in vitro *[14–16].

Multiple meta-analyses have indicated that NSCs are effective in animal models of ischemic stroke [17, 18]. However, the studies included used stem cells of different origins and different doses, routes, and timings of transplantation. Therefore, there were multiple confounding factors, and the conclusions drawn were insufficient to guide future animal and clinical studies. In addition, NSC-based therapies for ischemic stroke are also fairly controversial. For example, studies show that many inflammatory factors, cytokines, and reactive oxygen species are released during the acute and subacute phases of ischemic stroke, altering the microenvironment and ultimately leading to the destruction of NSCs [19, 20]. Conversely, findings from Darsalia et al. suggested that transplantation in the acute phase could improve the survival rate and therapeutic effect of NSCs [21]. Furthermore, Guan et al.’s results indicated that NSCs fail to improve motor function in rat models of ischemic stroke and instead result in an extremely high tumor incidence [22].

Meanwhile, there have been preliminary attempts to apply NSC therapy clinically. However, its suboptimal therapeutic effects and severe complications have limited the development of further large-scale clinical trials [23]. A major reason for the poor clinical success in patients with ischemic stroke is the lack of clarity surrounding the optimal parameters for NSC-based repair strategies (e.g., optimal source, dose, route, and timing of transplantation), which have received limited attention in preclinical studies. Meanwhile, given the relevant safety and ethics issues, it is even less feasible to explore these optimal parameters in clinical settings [13].

Therefore, there is a great need to identify optimal conditions for NSC-mediated ischemic stroke treatment based on preclinical studies, as this could reduce treatment risks, improve translation efforts, and guide clinical practice [3, 16, 24, 25]. To this end, we comprehensively reviewed published animal studies and explored the efficacy of different NSC-based strategies for ischemic stroke treatment. Using traditional and network meta-analysis, we attempted to identify optimal conditions for NSC therapies in order to provide a reference for future animal experiments and clinical research on ischemic stroke treatment.

## Methods

This systematic review and meta-analysis followed the Preferred Reporting Items for Systematic Reviews and Meta-Analyses (PRISMA) guidelines (Additional file 1) [26]. Although the study was not registered with the PROSPERO database or other comparable databases, we verified that no similar study has been registered before undertaking this study.

### Inclusion and exclusion criteria

#### Subjects

Animal models of ischemic stroke were included, no restrictions on species and modeling method.

#### Interventions

Neutral stem cells, with no restriction on sources.

#### Control

1) Positive control: Comparison of different sources, route, dose, timing of transplantation between NSCs; 2) Negative control: included Normal saline, PBS, Vehicle, Cultural medium, Blank, DMSO, DMEM

#### Outcome

1) modified Neurologic Severity Score (mNSS): The degree of injury was assessed by subjective scores in ischemic stroke animals. The higher the score, the worse the injury was.

2) Volume of cerebral infarction: The volume of cerebral infarct was measured by histological and / or imaging means.

#### Type of study

Randomized/open-label controlled studies were included, no restrictions on blind method.

#### Search strategy

Computer retrieval was conducted in PubMed, Ovid-Embase and Web of science, and the retrieval deadline was December 2021. The retrieval words: (ischemic stroke OR brain ischemia OR brain infarction OR cerebral infarction OR cerebral ischemic stroke OR intracranial ischemia OR cerebral arterial thrombosis) AND (neural stem cells OR neural precursor cell OR neural progenitor cell OR NSPC). See Additional file 2: Table 1 for the detailed retrieval process of each database.

### Literature screening and data extraction

Two trained researchers selected the papers. They extracted the data in strict accordance with the inclusion and exclusion criteria, and cross-checked them. In case of any disagreement, a third party was consulted to arrive at a consensus. Data were extracted according to a pre-established full-text data extraction checklist, which included:

(i) Basic information: author, year, and type of study; animal sex, age, body weight, sample size, and modeling methods; NSC type, source, transplantation dose, transplantation route, and transplantation timing; and intervention measures in the control group
(ii) Outcomes: modified neurological severity score (mNSS), volume of cerebral infarction

### Risk of bias assessment

To improve the quality of future animal studies and clinical trials, a critical evaluation of preclinical studies from the perspective of methodology, experimental design, implementation, and reporting is highly warranted. Based on SYRCLE’s risk of bias tool for animal studies [27], two trained researchers independently evaluated and cross-checked the inherent risk of bias in the included studies. They examined selection bias, implementation bias, measurement bias, follow-up bias, report bias, and other biases based on a list of 10 questions. Any difference in opinion was negotiated or resolved by consulting a third party. The answer to the assessment questions (tools) was either ‘‘yes” (indicating low risk of bias) or ‘‘no” (indicating high risk of bias). For unclear items, an ‘‘unclear” tag was assigned.

### Statistical analysis

Traditional and network meta-analyses were performed using the Bayesian model-based gemtc-0.14.3 software and stata16 software. The gemtc-0.14.3 software implements a reticular meta-analysis based on a Bayesian framework by applying a Markov Chain-Monte Carlo (MCMC) approach to priors and evaluations of the data. A consistency model was used for network meta-analysis, and *P* < 0.05 was considered statistically significant.

Inconsistency tests were performed using a node analysis model; a *P* value > 0.05 indicated no evidence of inconsistency between direct and indirect comparisons. The convergence of the network meta-analysis was examined using the potential scale reduced factor (PSRF), and a PSRF close to 1 indicated good convergence, demonstrating that the conclusions of the meta-analysis were reliable. Data were preprocessed using the network group command of stata16.0 software to draw a web plot for comparisons among various interventions and to detect publication bias. Meanwhile, the standardized mean difference (SMD) was used as the effect analysis statistic for traditional meta-analysis, and its 95% confidence interval (CI) was obtained for each effect size. The examination level was set at α = 0.05.

This study quantitatively analyzed the efficacy of NSCs for ischemic stroke treatment from both a macroscopic (mNSS) and histological (volume of cerebral infarction) level. Because all studies sacrificed animals at the end of the follow-up in order to measure the volume of cerebral infarct, traditional meta-analysis was performed only using data from the end of the follow-up period. Meanwhile, because all included studies used histological staining followed by software-based quantitative analysis for measuring the volume of cerebral infarct, the data were more objective. Hence, we conducted a network meta-analysis to explore the optimal parameters of NSC therapy. Most included studies reported the mNSS at 1 and 4 weeks; hence, traditional meta-analysis was performed for this parameter. However, as mNSS is an outcome based on subjective measurements, the final calculation of mNSS can differ based on the evaluator’s qualifications, experience, and understanding of evaluation entries, affecting the reliability of network meta-analysis findings [11]. Accordingly, we did not perform a network meta-analysis for mNSS.

## Results

### Results of literature search

**Fig. 1:**
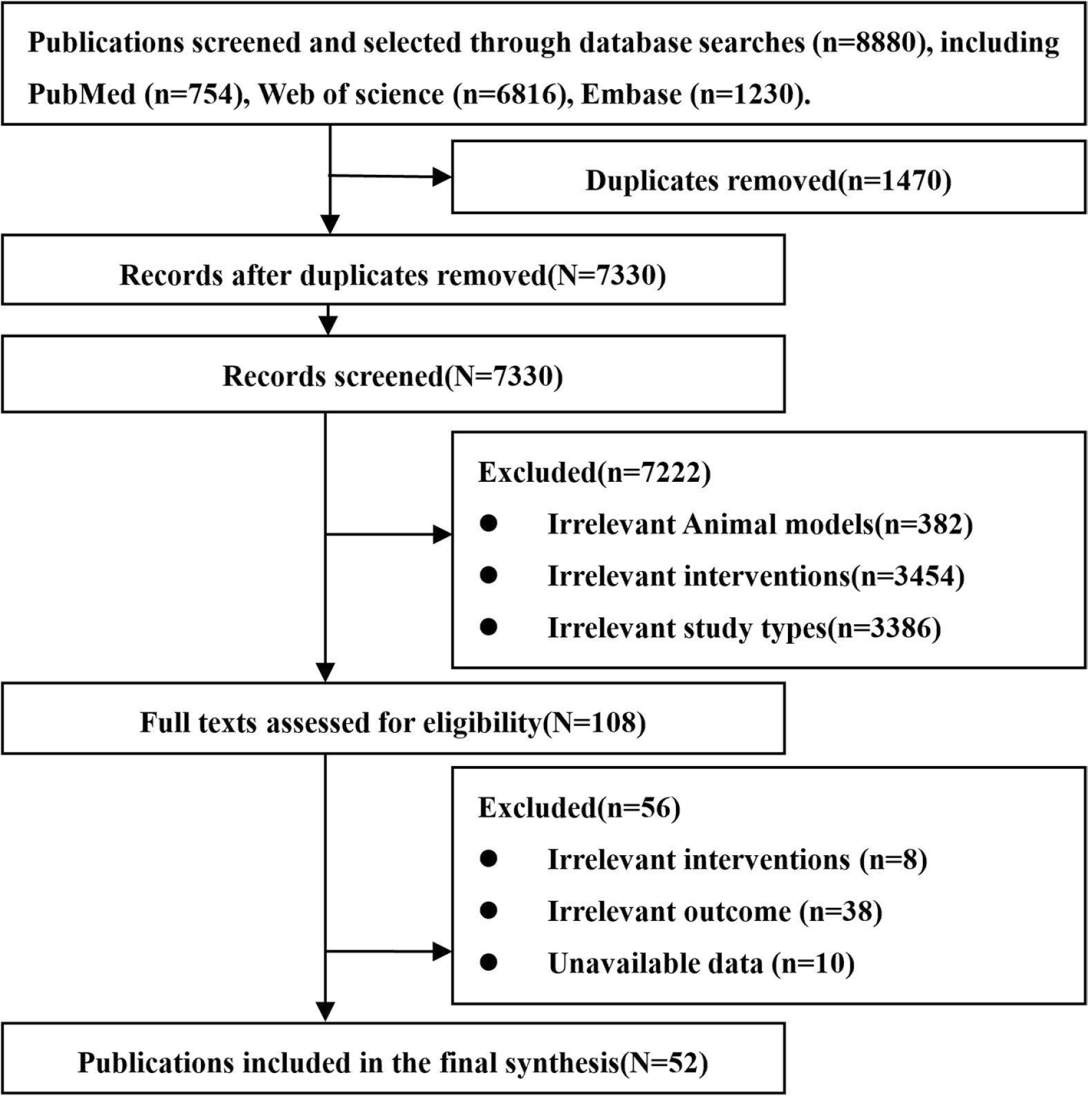
Flowchart of literature-screening process

A total of 8,880 references were searched through the specified databases. Repetitions were excluded. Further, studies with unrelated animal models (brain trauma, human ischemic stroke with diabetes, Alzheimer’s disease, Parkinson’s disease, Huntington’s disease, etc.); studies with unrelated interventions (other types of stem cells, stem cell-derived exocrines, growth factors, etc.); studies with irrelevant outcome indicators (no mNSS or volume of cerebral infarction reported); studies in which data could not be obtained; and non-research papers (reviews, conference abstracts, letters, and editorials, etc.) were also excluded. Finally, 52 animal studies in which NSCs were used for treating ischemic stroke were included. The flow chart of the literature screening process is presented in Fig. 1.

### Basic information of included studies

All the 52 animal studies included were randomized controlled trials. The animal models included Wistar (*n*=9 studies) and Sprague–Dawley (SD) rats (*n*=29 studies), weighing 200–360 g and aged 7 weeks to 24 months; and C57BL6/J (*n*=7 studies), Kunming (*n*=1 study), Fisher 344 (*n*=1 study), ICR (*n*=3 studies), and Albino mice (*n*=1 study), weighing 20–25 g and aged 1–12 weeks. Further, one study examined Mongolian gerbils weighing 60–72 g and aged 6–22 weeks. The age of the animals was not reported in 16 studies, and the weight of the animals was not reported in eight. All studies used animals that were male, except for one that did not report the sex of the animals and three that used both male and female mice. The modeling procedures included the use of endothelin-1 to constrict the middle cerebral artery (MCA, *n*=2 studies), the use of wire plugs to occlude the MCA (*n*=38 studies), the use of vascular clipping to occlude the MCA (*n*=1 study), the use of electrocoagulation to block the MCA (*n*=1 study), and photooxidation to induce cortical and striatal damage and cause ischemic stroke (*n*=3 studies). Seven studies did not report the specific modeling process.

The sources of NSCs included allogeneic and xenogeneic tissues. The routes of transplantation included the striatum (*n*=18 studies), ventricles (*n*=7 studies), caudate nucleus (*n*=1 study), infarct region (*n*=5 studies), hippocampus (*n*=3 studies), cerebral cortex (*n*=6 studies), tail vein (*n*=10 studies), femoral vein (*n*=1 study), and jugular vein (*n*=1 study). The timing of transplantation ranged from immediately after modeling to 3 weeks postoperatively. The number of transplanted cells ranged from 5×10^4^ to 5×10^6^ NSCs, but this number was not reported in three studies. Negative controls included DMEM (*n*=1 study), medium (*n*=5 studies), PBS (*n*=32 studies), vehicle (*n*=7 studies), blank (*n*=2 studies), and saline (*n*=5 studies). The basic information of the study subjects is summarized in Additional file 2: Table 2.

### Risk of bias assessment

**Fig. 2:**
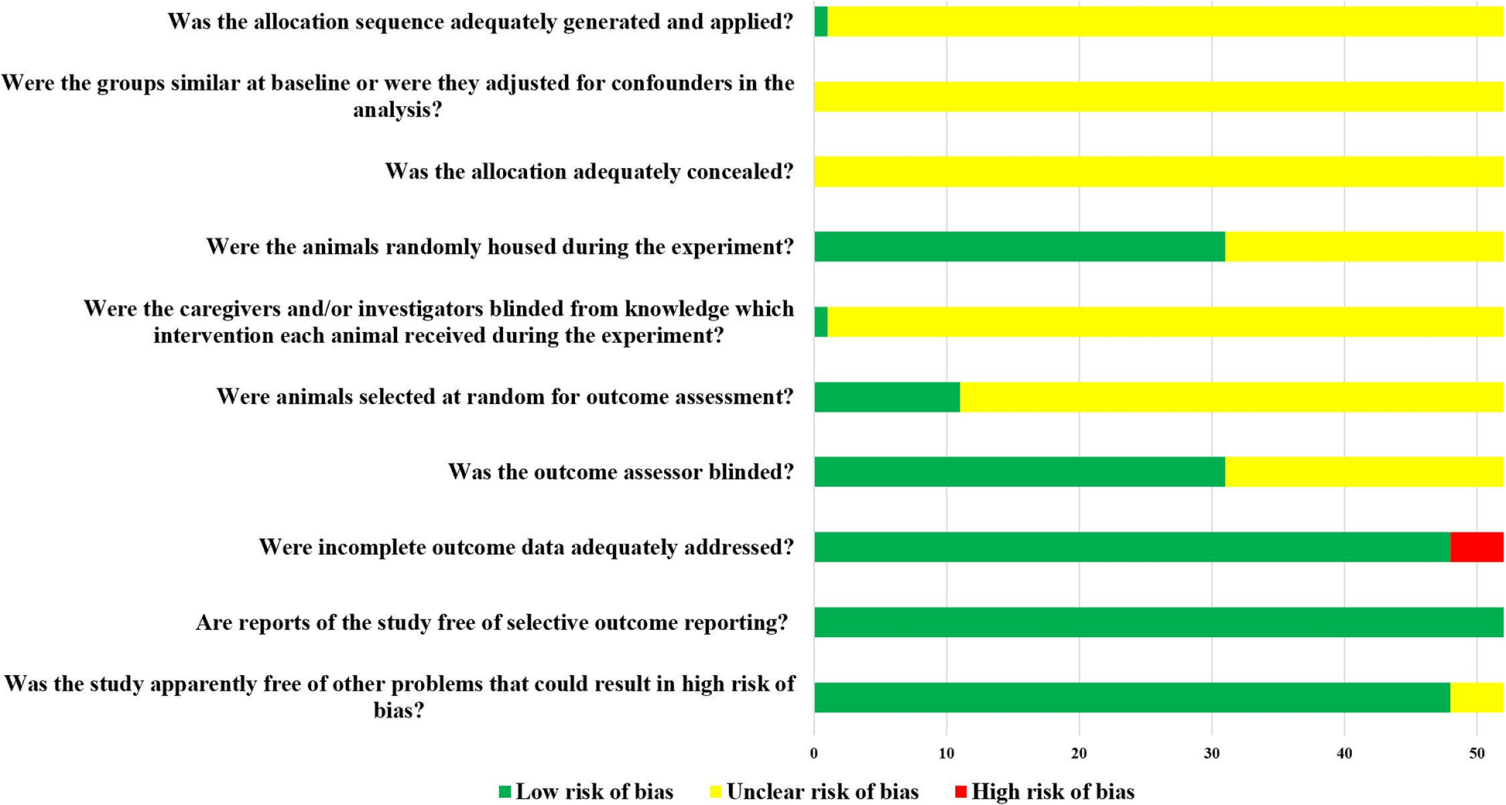
Risk of bias for each item of the SYRCLE checklist in all included studies. Each risk of bias item is presented as a percentage across all included studies, indicating the proportion of different levels of bias risk for each item

Of the 52 included studies, only one study reported the randomization of animals using the random numbers table. However, it did not report whether concealed grouping was implemented. All studies reported the matching of baseline characteristics such as the age, sex, and body weight of animals among groups. In 31 studies, animals were randomly housed during experiments. Only one study reported that animal breeders and/or researchers were blinded to the study groups, although it did not report the specific processes. Only 11 studies reported that animals were randomly selected for evaluation. Moreover, 31 studies reported the implementation of blinding procedures for the evaluators. In four studies, animals were withdrawn during the experimental procedure, although the impact of withdrawals on the results was not examined. While the research protocol was not available for any of the studies, all expected results were clearly reported. The risk of bias assessment for all studies is detailed in Fig 2.

### Traditional meta-analysis of volume of cerebral infarction

Of the 52 included studies, 31 studies reported the volume of cerebral infarction. Meta-analysis using a random effects model showed that animals in the NSCs group had a significantly lower cerebral infarct volume than those in the negative control group (SMD=-2.05[-2.73, -1.36]). Details are provided in Additional file 2: Figure 1.

### Traditional meta-analysis of mNSS

In total, 38 of the 52 included studies reported the mNSS of animals after NSC transplantation. Meta-analysis using a random effects model revealed a significantly lower mNSS in animals treated with NSCs than in animals from the negative control group at 1 week (SMD = -2.44 [-3.19, -1.69]; details in Additional file 2: Figure 2) and 4 weeks after transplantation (SMD=-3.53[-4.51, -2.45]; details in Additional file 2: Figure 3).

**Fig. 3:**
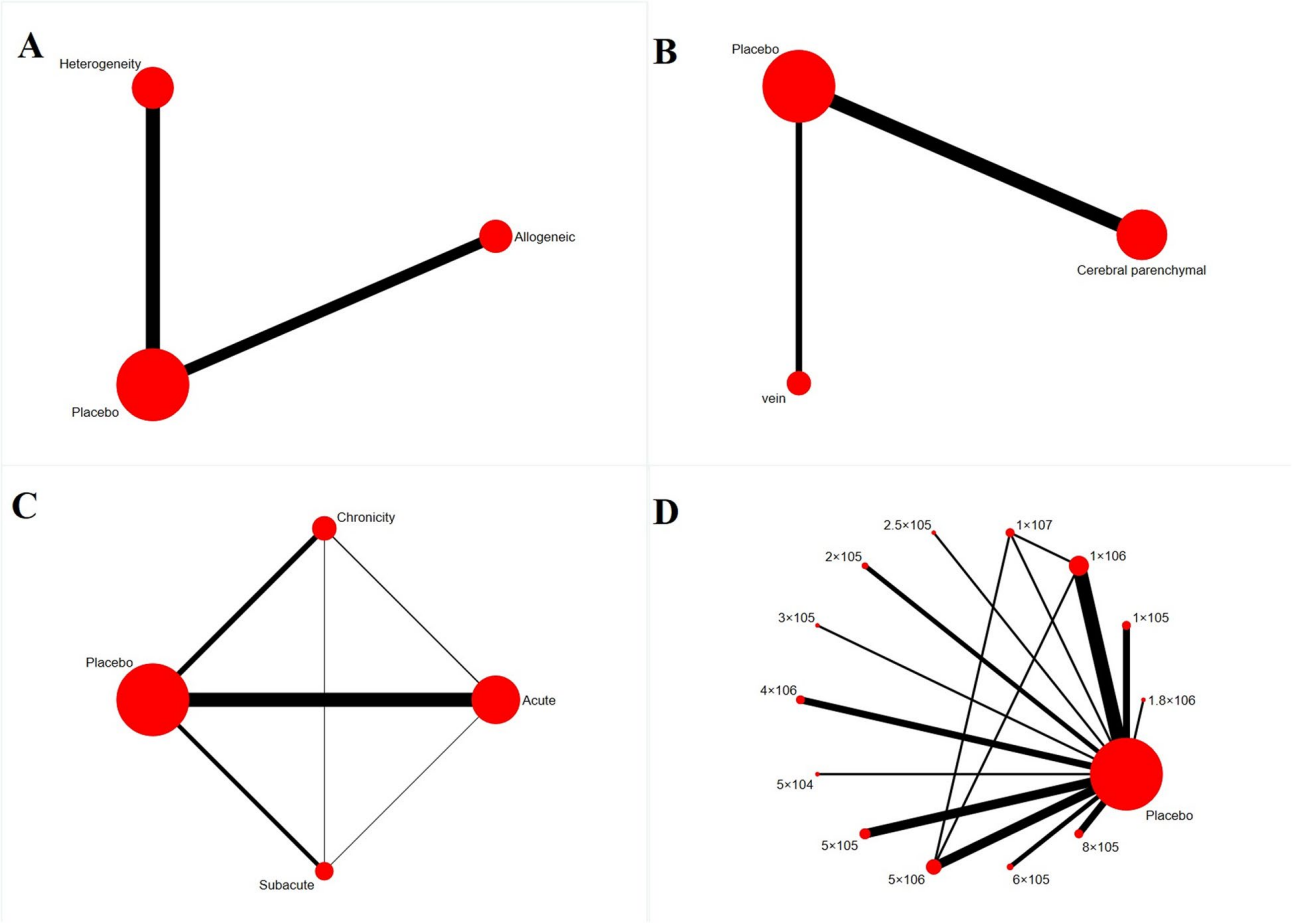
Evidence network diagram (circle size represents sample size involved; thickness of line segment represents number of studies involving both interventions; **A**. Source of NSCs; **B**. Route of transplantation; **C**. Timing of transplantation; **D**. Dose of transplantation)

### Network meta-analysis of volume of cerebral infarction

#### Source of NSCs

**Fig 4.**
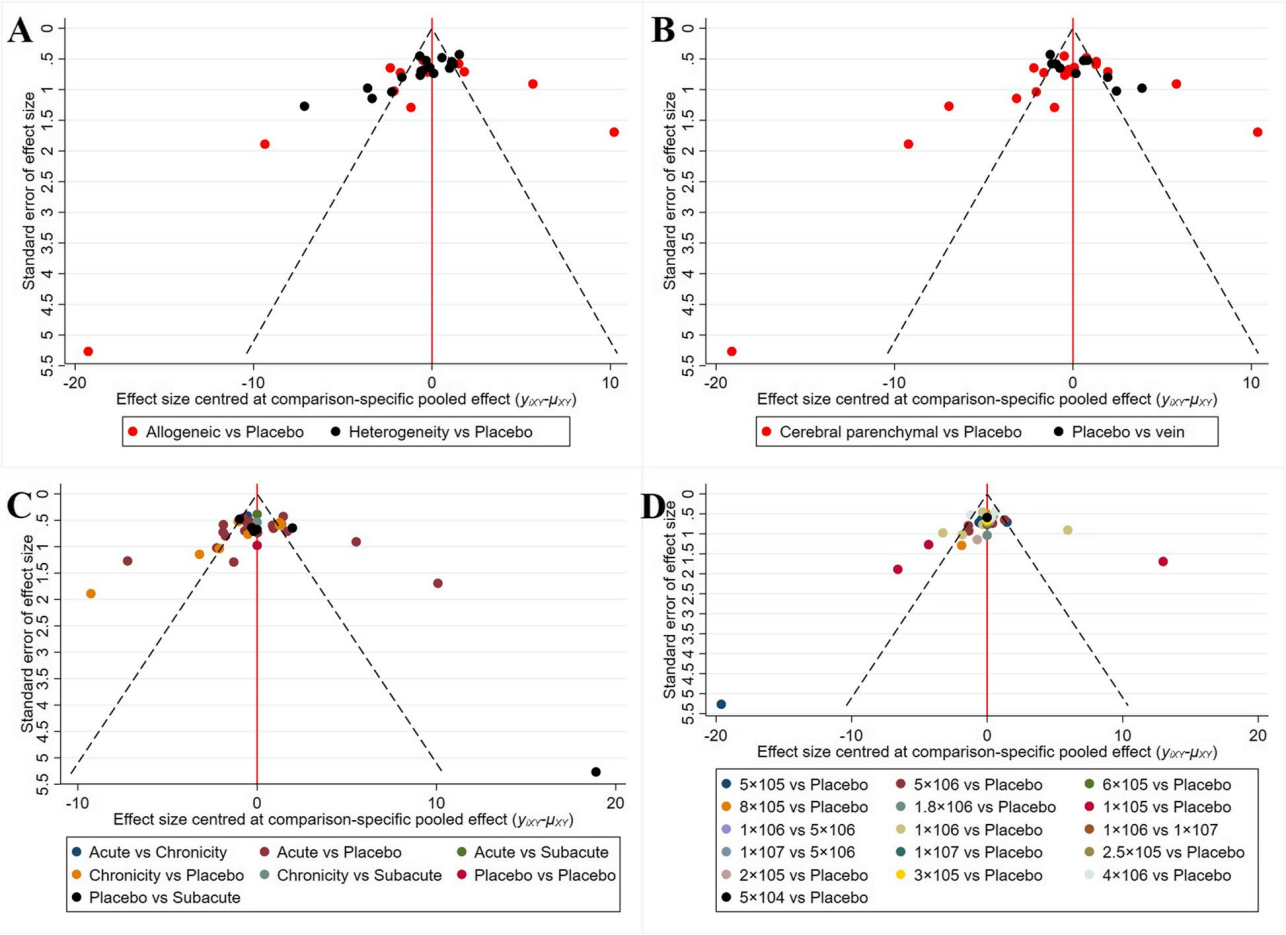
Comparison–correction funnel plots (**A**. Source of NSCs; **B**. Route of transplantation; **C**. Timing of transplantation; **D**. Dose of transplantation)

The source of NSCs in the included studies could be divided into two categories: cells from different animals of the same breed (Allogeneic) or cells from a different species (Xenogeneic). The evidence network showed no evidence of direct contrast between NSCs of different sources (Fig. 3A). Network meta-analysis showed that there was no significant difference in the ability of NSCs from different sources to reduce infarct volume (SMD= -2.34 [-18.38, 13.44]). The comparison-correction funnel plot was asymmetric, suggesting that there may be publication bias and small sample effects, as shown in Fig. 4A. Further, the ranking results showed that the ability of allogeneic NSCs to reduce cerebral infarct volume was superior to that of xenogeneic stem cells (Rank: allogeneic=38% < xenogeneic=61%. We performed a network meta-analysis based on the volume of cerebral infarction. Hence, a lower ranking result indicated a lower likelihood of cerebral infarction and a better treatment effect.

Although the best source of NSCs was the brain tissue of allogeneic animals, four sources (embryo, fetus, neonate, and adult) were included. Further network meta-analysis showed that there was no significant difference in the ability of these four types of NSCs to reduce the volume of cerebral infarction. However, the ranking results showed that the ability to improve the volume of cerebral infarction was highest in embryonic NSCs (2%), followed by neonatal (5%), fetal (31%), and adult NSCs (33%).

#### Route of transplantation

The route of cell transplantation in the included studies could be classified into intracerebral and intravenous transplantation. The evidence network showed that direct contrast between NSCs transplanted using different routes was currently lacking (Fig. 3B). Network meta-analysis showed that there was no significant difference between NSCs transplanted intracerebrally and intravenously in reducing the volume of cerebral infarction (SMD=-2.56 [-20.45, 14.35]). The comparison–correction funnel plot was asymmetric, suggesting that there may have been publication bias and a small sample effect (Fig. 4B). However, the ranking results showed that the therapeutic effect of intracerebral NSC transplantation was superior to that of intravenous NSC transplantation (Rank: cerebral parenchymal=0 < vein=6%).

#### Timing of transplantation

We divided the timing of transplantation after animal modeling into three stages: acute phase (≤ 1 day), subacute phase (1–7 days), and chronic phase (≥ 7 days). The evidence network showed that direct comparisons could be made between studies with different timings of transplantation, but most studies involved comparisons between different timings of treatment and the placebo, as detailed in Figure 3C. The results of network meta-analysis showed that there was no significant difference between different transplantation timings of NSCs in reducing the volume of cerebral infarction, as detailed in Table 1. The comparison–correction funnel plot was asymmetric, suggesting that there may be publication bias and small sample effects, as shown in Fig. 4C. However, the ranking results showed that the transplantation of NSCs in the acute phase was more effective than that in the subacute and chronic phases (Table 2).

**Table 1:** Network meta-analysis results for timing of transplantation

Acute			
6.84 (-14.76, 28.82)	Subacute		
11.81 (-8.55, 31.47)	4.74 (-20.89, 30.02)	Chronic	
-11.16 (-22.46, -0.18)	-18.17 (-37.57, -1.35)	-22.87 (-40.39, -5.37)	Placebo

**Table 2:** Ranking results for optimal timing of transplantation

**Timing of transplantation**	**Rank 1**	**Rank 2**	**Rank 3**	**Rank 4**
Acute	0.01	0.65	0.28	0.05
Subacute	0.03	0.21	0.43	0.33
Chronic	0.02	0.08	0.29	0.62
Placebo	0.94	0.06	0	0

#### Dose of transplantation

No study has so far delineated the optimal dose of transplantation for NSCs. Therefore, we examined the optimal number of transplanted NSCs using network meta-analysis without using any dose range. The results of the evidence network showed that direct contrast between NSCs of different doses was currently lacking, as detailed in Fig. 3D. In order to avoid the influence of factors such as body weight and species of experimental animals on the results, the experimental animals were divided into two subgroups: rats and mice. The results of network meta-analysis revealed no significant difference in the reductions of cerebral infarction volume among different transplantation doses of NSCs, as detailed in Additional file 2: Tables 3 and 4. The comparison-correction funnel plot was asymmetric, suggesting that there could be publication bias and small sample effects (4D). The ranking results showed that in the rat group, the transplantation dose of 1 × 10^6^ and 1.8 × 10^6^ of NSCs could have the best effect in reducing the volume of cerebral infarction (Additional file 2: Table 5). In mice, the transplantation dose of 1–5×10^5^ NSCs appeared to have the best effect in reducing the volume of cerebral infarction (Additional file 2: Table 6).

#### Sensitivity analysis of cerebral infarct volume

**Fig. 5:**
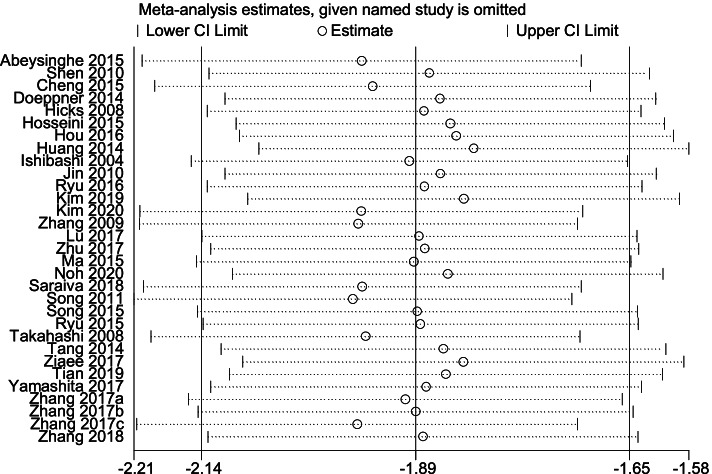
Sensitivity analysis results of cerebral infarct volume

To explore the impact of single studies and ensure the reliability of the meta-analysis results, we performed a sensitivity analysis of infarct volume. The results of sensitivity analysis showed that the included studies had good consistency (Fig. 5).

## Discussion

In recent years, there have been fundamental advances in the understanding of stroke pathophysiology, optimization of animal models, imaging techniques, and methodology [28]. However, very few stroke patients benefit from NSC therapy [29]. Therefore, it is essential to fully explore the therapeutic potential of NSCs and the optimal parameters of NSC therapy in animal studies to reduce the risk in clinical trials and guide clinical practice [30]. Therefore, we comprehensively analyzed the therapeutic effects of NSCs in animal studies using traditional and network meta-analysis methods while exploring optimal stem cell-based treatment strategies.

### Summary of evidence

#### Results of traditional meta-analysis

Consistent with previous meta-analyses, our results showed that the ability of NSCs to reduce the mNSS and volume of cerebral infarct was significantly superior to that of the negative control in animal models of ischemic stroke. Although traditional meta-analyses have demonstrated that NSCs have a promising role in treating ischemic stroke, they analyzed stem cells of different sources and different doses, routes, and timings of transplantation together. These comparisons of “comprehensive” findings were challenging to replicate in clinical settings and were not strong enough to guide clinical practice. Therefore, we explored the optimal parameters of NSC-based treatment strategies for ischemic stroke through network meta-analysis based on traditional meta-analysis in order to provide a reference for clinical practice.

#### Best source of NSCs

Autologous stem cells are an ideal source of stem cells because they are associated with lower rates of immune rejection than allogeneic or xenogeneic stem cells [31]. However, the therapeutic window for patients with ischemic stroke is limited, making it difficult to obtain sufficient autologous stem cells in a short period. However, a clinical cell bank that contains stem cells of allogeneic or xenogeneic origin that can be transplanted at any time after stroke, including the first few hours, could help in rescuing brain function in stroke patients [32]. Therefore, to assist with clinical feasibility, most animal studies use cells from allogeneic animals (allogeneic stem cells) or human tissues (xenogeneic stem cells). In this study, network meta-analysis showed that the therapeutic effect of allogeneic stem cells was better than that of xenogeneic stem cells. This could be because the risk of graft rejection is lower with allogeneic stem cells [3]. Therefore, we consider allogeneic stem cells to be the best source of stem cells, as they can prevent both the safety and ethics issues associated with xenogeneic stem cells. Moreover, they can circumvent the problems caused by the shortage of autologous stem cells. However, the tolerance of allogeneic stem cells can greatly hinder their function, and this needs to be explored in further studies. In addition, although NSCs can be isolated from the cerebral cortex, striatum, hippocampus, olfactory bulb, ventricles, and other tissues of the central nervous system (CNS), the proliferation and differentiation potential of derived NSCs differs according to the developmental stage [33]. Hence, we further compared the specific sources of allogeneic stem cells and found that the therapeutic potential of embryonic tissue-derived NSCs was superior to that of NSCs derived at other stages. This is mainly related to the gradual decrease in NSCs with the maturation of the CNS and acquisition of quiescence. NSCs from embryonic brain tissue are in an active state and have stronger self-replication and proliferation ability [34].

#### Optimal route of NSC transplantation

The route of exogenous stem cell delivery into diseased brain tissue is important for improving the efficiency of stem cell transplantation and repair. Intravenous administration, the simplest and safest traditional mode of administration, has been widely used for the treatment of various diseases. However, intravenously transplanted NSCs tend to aggregate in nontarget areas (the liver or lungs), and it is difficult to achieve good therapeutic delivery of cells through the blood–brain barrier. This reduces the efficiency of stem cell migration to the region of the infarct [35, 36]. In addition, the potential adverse effects of venous transplantation, such as tumor formation and vascular embolization, have also raised strong concerns among clinicians and patients [3]. In contrast, intracerebral transplantation enables the precise transplantation of stem cells around the lesion. This increases the number of transplanted cells in and around the infarct, which enhances cell survival and differentiation, thereby promoting tissue damage repair. Our network meta-analysis showed that the intracerebral transplantation of NSCs had a better therapeutic effect than did intravenous transplantation. This is because with intracerebral transplantation, stem cells are directly introduced into the target area, resulting in a high cell transplantation and survival rate[37]. It is worth noting that although NSCs show a good repair effect in preclinical studies, unlike animals, human patients with ischemic stroke do not have an open wound. Hence, stem cells cannot be directly transplanted to the site of cerebral infarction in humans. In clinical settings, invasive intracerebral transplantation may further aggravate brain injury and also lead to serious complications, such as intracranial hypertension, subdural hematoma, and thrombophlebitis [3, 23, 38]. Therefore, the clinical risk with intracerebral transplants is higher due to invasive manipulation and severe complications. Hence, whether this approach is feasible for patients remains controversial [39]. Some studies have shown that the transplantation of stem cells to the brain can disrupt the physiological balance in the damaged area and disrupt the effect of inflammatory factors, thereby reducing the survival and differentiation capacity of transplanted cells [40]. Therefore, the efficacy and safety of intracerebral NSC transplantation must be explored in the future to promote the clinical translation of experimental results obtained from animal studies. In addition, intra-arterial transplantation has been shown to be a more direct route for transferring stem cells to the lesion site and achieving improved stem cell engraftment efficiency [41, 42]. Intranasal transplantation has also been shown to bypass the blood–brain barrier and deliver stem cells to the lesion [43]. However, studies related to arterial or intranasal transplantation are limited, and the efficacy and safety of these routes need to be evaluated in future studies.

#### Optimal timing of NSC transplantation

The environment within the CNS changes dramatically with the course of ischemic stroke, and the pathophysiology of ischemic stroke is complex. Therefore, the timing of NSC transplantation largely determines the final therapeutic outcomes [36]. However, the period of ischemic stroke injury in animal models is not clear. Currently, the timing of stem cell transplantation mainly depends on the investigators conducting the animal studies and ranges from hours to days after stroke induction [44]. Indeed, the survival and migration of transplanted NSCs are significantly affected by the inflammatory response associated with stroke. Previous studies have shown that after ischemic stroke, many granulocytes, T cells, monocytes/macrophages, and other inflammatory cells accumulate in the ischemic brain area. These cells release TNF-a, IL-1b, IL- 6, IL-20, IL-10, and TGF-b, thus damaging the exogenous NSCs implanted during the early stage [45, 46]. In contrast, Wang et al. showed that animals receiving stem cell transplantations within 3 and 24 h post-ischemia achieve better histological and behavioral outcomes than those receiving transplantations on day 7 [47]. This is mainly because the growth factors released at early stages can improve the survival, differentiation, and/or integration of transplanted cells. In the subacute or chronic phase, the release of growth factors is low. Moreover, the glial scars formed by astrocytes during these stages may severely limit axon regeneration and synapse formation, thereby hindering the function of exogenous cells. Although previous studies have demonstrated that stem cell transplantation can be effective at different time-points, the magnitude of the therapeutic effect is controversial. The results of our network meta-analysis showed that stem cell transplantation performed in the acute phase is superior to that performed in the subacute and chronic phases. One possible reason is that stem cell transplantation in the acute phase can reduce the production of reactive oxygen species and proinflammatory factors during the early stroke process. It can enable the replacement of damaged NSCs, thereby exerting neuroprotective effects[3]. Stem cells transplanted in the acute phase also help in neuron rescue in the ischemic penumbra. They act on inflammatory cells early on and activate inflammatory cascades, thus promoting the recovery of neurological function [48]. Studies have shown that the injury caused by stroke mainly leads to a substantial increase in the number of microglia in the injured area—a result of local microglial proliferation and macrophage infiltration from the blood [49, 50]. Microglia have positive effects on neurogenesis, but their negative effects are more pronounced [51]. Microglial activation is low during the first 48 h after stroke, and then gradually increases; this higher rate of activation can be maintained for about 6 weeks [21]. Therefore, the early transplantation of NSCs after stroke can reduce the exposure of exogenous stem cells to the inflammatory environment. Meanwhile, clinically, the administration of stem cells via a catheter immediately after intravascular perfusion therapy in ischemic stroke patients can prevent the injury induced by a second surgery. In addition, studies have shown that endogenous NSCs migrate to vascular remodeling areas within 24 hours after stroke and promote angiogenesis by secreting vascular endothelial growth factor [52–54]. Unfortunately, endogenous NSCs cannot completely repair nerve injury because they are limited in number and have insufficient regenerative capacity [55, 56]. Therefore, the transplantation of exogenous NSCs at an early stage can also promote endogenous NSC-mediated injury repair [21, 37].

#### Optimal dose of NSCs

The number of transplanted stem cells has a significant effect on ischemic stroke lesion volume and motor function recovery, and a dose–response relationship exists within a certain range [57].

However, some studies show that the relationship between the neurological function benefits and number of transplanted stem cells may be inverse U-shaped. An optimal number of transplanted stem cells exists. An excess or insufficient number of transplanted stem cells can alter therapeutic effects [58]. The results of our network meta-analysis were consistent with these findings, showing that more is not always better when it comes to transplantation doses. In mice, 1–5×10^5^ NSCs had the best therapeutic effect, while in rats, 1×10^6^ or 1.8×10^6^ transplanted NSCs had the best effect. This is also in line with the results obtained by Huang et al, who divided animals into three subgroups based on transplantation dose (≤1×10^6^, 1–5×10^6^ and >5×10^6^), and found that low-dose transplantation (≤1×10^6^) is better than the higher doses in improving function [17]. This could be because when the NSCs are low in number, they can directly migrate to the lesion area, instead of forming cell clusters and causing microembolization and tumor formation, thereby aggravating ischemic brain injury [59].

In conclusion, although the number of transplanted stem cells can alter the therapeutic effect, an excessively high transplant dose can cause blood vessel occlusion, thereby reducing the therapeutic effect. Exceeding the optimal dose threshold can also saturate damaged brain tissue, resulting in the low availability of nutrients for transplanted cells and thereby reducing the survival rate of exogenous cells [21]. In addition, according to Stem Cell Therapy as an Emerging Paradigm for Stroke (STEPS), when studying the transplantation dose of stem cells, the dose should be based on the body weight of the experimental animal [60]. Therefore, we divided the experimental animals into a rat group and a mouse group for subgroup analysis according to differences in body weight and genetic background. We found that the optimal transplantation dose of stem cells in rats was much higher than that in mice. This also shows that differences in body weight and genetic background can affect the therapeutic effect of stem cells. However, due to the limited availability of detailed information, such as the baseline characteristics of animals, and the limited number of studies, we were unable to analyze other influencing factors in more detail.

### Quality of evidence

#### Heterogeneity in included studies

Eight different varieties of rats and mice were included in the study. Moreover, the age of the animals varied from 1 week to 24 months and their body weight ranged from 20 to 360 g. Thus, there were large differences in the baseline characteristics of the animals. Further, the heterogeneity of the baseline characteristics of model animals in different studies may have led to significant differences in the responses to NSCs. For example, the absolute value of cerebral infarct volume in rats may be much larger than that in mice. Furthermore, different studies reported different outcomes for cerebral infarct volume. Some studies reported the absolute value of cerebral infarction, while some reported the percentage of cerebral infarct volume occupied in the ipsilateral or contralateral hemisphere or in the whole brain. In addition, there was also great variability in the time points at which cerebral infarct volume was measured. Given the heterogeneity present in the included studies, we performed data analysis using a random effects model and used SMD values. Therefore, the results and conclusions drawn need to be interpreted and treated with caution.

#### Insufficient intrinsic authenticity of included studies

Although the baseline characteristics of the animals were balanced, the method of randomization was unclear for 98.08% (51/52) of the studies. Moreover, none of the studies reported whether concealed grouping was implemented. Therefore, there was a certain selection bias. In addition, only one study reported the blinding of animal feeders/investigators to study groups, and only 59.62% (31/52) of studies implemented evaluator blinding protocols. Therefore, the included studies had a certain level of implementation bias and measurement bias. In addition, for outcome measures, especially those requiring subjective judgment, the qualification of the evaluators and the consistency with different animal measurement standards can affect results. Moreover, the accuracy and scientific validity of the measures can affect results to different degrees, although blinding can prevent the impact of measurement bias on the outcome [61]. However, none of the 52 studies included in our systematic review reported the qualification of the evaluators and their standards and the specific measurement process at the time of the outcome measurement. Therefore, future studies should comprehensively report specific experimental implementation details in order to improve the reproducibility and reliability of animal experimental results. Although all studies clearly reported their findings, the study protocols were not reported. Hence, the possibility of selective outcome reporting, which can lead to publication bias, cannot be ruled out [62]. Therefore, a reporting and registration system for animal studies should be encouraged to further improve the reproducibility and reliability of experimental results [63].

#### Insufficient external authenticity of included studies

External authenticity refers to the extent to which clinical results can be reproduced repeatedly in the target population and the common population [64]. The external authenticity of several aspects needs to be considered while translating animal experimental results to clinical trials. 1) In most animal studies, stem cells are directly transplanted into the infarct area. However, it is difficult to do this clinically. Therefore, whether the benefits observed in animal studies can be translated to the clinic needs to be interpreted with caution. 2) Imaging techniques can be used in animal studies to explore specific repair mechanisms via fluorescence-labeled cells. However, it is difficult to use these techniques in clinical studies due to ethical limitations [65]. 3) Clinically relevant endpoints related to higher neurological function, such as cognition and learning and memory are challenging to measure in animal models [66]. 4) The safety of drugs or therapies is a prerequisite for their clinical application and is even more important than their effectiveness [67]. The studies included in our systematic review only measured the effectiveness indicators of stem cell therapy, and few studies reported safety indicators, such as the tumorigenicity of stem cells, immune rejection, and allergic reactions [68]. 5) Various sources of NSCs are used in animal studies. NSCs for these studies are obtained from autologous neural tissue or allogeneic/xenogeneic tissues. However, the availability of autologous or allogeneic human sources of NSCs are limited, and their extraction is associated with strong ethical concerns [69, 70].

#### Strengths and limitations of our study

The key strengths of our study are as follows. 1) Based on animal studies, the real effects and limitations of NSC-based treatments in ischemic stroke were systematically evaluated and analyzed, and the current problems and areas of improvement were highlighted. 2) Our network meta-analysis revealed optimal parameters for NSC-based treatment. To our knowledge, this study is the first to elucidate these parameters, and it is therefore valuable for designing relevant animal and clinical studies in the future. 3) Based on the internationally recognized SYRCLE bias risk assessment tool, the internal risk of bias was strictly evaluated in the included studies. The problems in the design and implementation of the animal studies in this field were identified, and suggestions for improving their quality were provided.

Nevertheless, the systematic review also had several limitations. 1) Owing to the limitations of the original data, we combined the data on cerebral infarct volume measured at different time points for the final analysis. This reduced the consistency and reliability of the meta-analysis. 2) When we analyzed the optimal sources of NSCs and the optimal doses, routes, and timings of NSC transplantation, we had to assume that no other factors influenced the results. For example, we did not consider the effect of timing and route of transplantation on outcomes when analyzing the optimal dose. This could have reduced the reliability of the conclusions. 3) The rationale of this practice is worth exploring, as we classified stem cells grafting into the cortex, striatum, ventricles, and other brain regions as intracerebral transplantation. 4) Because the condition of ischemic stroke changes quickly, the delineation of injury timing is challenging. Although we divided the phases of injury based on previous studies, the rationale of this division warrants further exploration [66]. 5) Because the source of heterogeneity could not be accurately identified, we used a random effects model for meta-analysis, making our conclusions more conservative; 6) Only Chinese and English databases were searched, which could have led to a certain language bias. 7) The grey literature and conference abstracts were not searched, which may have led to some publication bias.

## Conclusions

Through a comprehensive analysis of the 52 included studies, we found that NSCs significantly reduced the mNSS and volume of cerebral infarction in animal models of ischemic stroke. Our network meta-analysis showed that allogeneic embryonic tissue was the optimal source of NSCs, intracerebral transplantation was the optimal route of transplantation, and the acute phase was the optimal timing of transplantation. In the mouse model, the transplantation dose of 1–5×10^5^ showed the best therapeutic effect, whereas in the rat model, the best effect was observed with a transplantation dose of 1×10^6^ and 1.8×10^6^ NSCs.

There are significant differences between animal models of ischemic stroke and clinical patients. However, it is still important to fully explore the true efficacy and optimal therapeutic conditions of NSC-based treatments for ischemic stroke in preclinical studies to improve the quality of clinical trials. Through a comprehensive analysis of evidence quality, internal authenticity, and external authenticity, our study highlighted the problems in current animal studies on the treatment of ischemic stroke using NSCs. These problems were related to randomization, allocation concealment, blinding method, and measurement and reporting of the results, which may affect the authenticity and reliability of animal experimental findings. Therefore, in future animal studies, there is a need to further standardize study protocols and reporting parameters. This will improve the quality of animal studies and reduce patient risk when preclinical findings are translated to clinical settings.

## Supplementary Information

**Additional file 1.** 

**Additional file 2: Table 1. **Search strategies.** Table 2. **Basic information of included studies. **Table 3.** Results of network meta-analysis among neural stem cells with different transplantation doses in rats. **Table 4.** Results of network meta-analysis among neural stem cells with different transplantation doses in mice. **Table 5.** Ranking results between different doses of neural stem cells transplantation in rats. **Table 6.** Ranking results between different doses of neural stem cells transplantation in mice. **Figure 1.** Traditional meta-analysis results of cerebral infarction volume. **Figure 2.** Traditional meta-analysis results of the first week of mNSS. **Figure 3.** Traditional meta-analysis results of the fourth week of mNSS. 

## Data Availability

Datasets are available through the corresponding author upon reasonable request.

## Abbreviations

r-tPA: Recombinant tissue-type plasminogen activator
NSCs: Neural stem cells
mNSS: Modified Neurologic Severity Score
MCMC: Markov chain-Monte Carlo
PSRF: Potential scale reduced factor
SMD: Standardized mean difference
MCA: Middle cerebral artery
