# Supplementary material for: Optimal therapeutic conditions for the neural stem cell-based management of ischemic stroke: a systematic review and network meta-analysis based on animal studies
Source: BMC Neurol. 2022 Sep 13;22:345. doi: 10.1186/s12883-022-02875-z (PMC9469626; doi:10.1186/s12883-022-02875-z)
Supplement: Supplementary file 2 — Additional file 2: Table 1. Search strategies. Table 2. Basic information of included studies. Table 3. Results of network meta-analysis among neural stem cells with different transplantation doses in rats. Table 4. Results of network meta-analysis among neural stem cells with different transplantation doses in mice. Table 5. Ranking results between different doses of neural stem cells transplantation in rats. Table 6. Ranking results between different doses of neural stem cells transplantation in mice. Figure 1. Traditional meta-analysis results of cerebral infarction volume. Figure 2. Traditional meta-analysis results of the first week of mNSS. Figure 3. Traditional meta-analysis results of the fourth week of mNSS. [file 12883_2022_2875_MOESM2_ESM.docx]

**Optimal therapeutic conditions for the** **neural stem cell-based management of ischemic stroke: a systematic review and network meta-analysis based on** **animal studies**

**Table 1: Search strategies**

| **Web of science**  (TS= (neural stem cells or neural precursor cell or neural progenitor cell or NSPC)) AND ((TS= (ischemic stroke OR brain ischemia OR brain infarction OR cerebral infarction OR cerebral ischemic stroke OR intracranial ischemia OR cerebral arterial thrombosis)) 6816  **PUBMED**  #1: "Neural Stem Cells"[MeSH Major Topic] 8992  #2: "neural stem cells"[Title/Abstract] OR "neural precursor cell"[Title/Abstract] OR "neural progenitor cell"[Title/Abstract] OR "NSPC"[Title/Abstract] 13230  #3: #1 OR #2 17606  #4: "Ischemic Stroke"[MeSH Major Topic] OR "Brain Ischemia"[MeSH Terms] OR "Brain Infarction"[MeSH Major Topic] OR "Cerebral Infarction"[MeSH Terms] 117522  #5: "ischemic stroke"[Title/Abstract] OR "brain ischemia"[Title/Abstract] OR "brain infarction"[Title/Abstract] OR "cerebral infarction"[Title/Abstract] OR "cerebral ischemic stroke"[Title/Abstract] OR "intracranial ischemia"[Title/Abstract] OR "cerebral arterial thrombosis"[Title/Abstract] 72865  #6: #4 OR #5 152248  #7: #3 AND #6 754  **EMBASE**  #1: (neural stem cells or neural precursor cell or neural progenitor cell or NSPC).mp. [mp=title, abstract, heading word, drug trade name, original title, device manufacturer, drug manufacturer, device trade name, keyword heading word, floating subheading word, candidate term word] 18358  #2: exp *neural stem cell/ 11566  #3: 1 or 2 22628  #4: (ischemic stroke or brain ischemia or brain infarction or cerebral infarction or cerebral ischemic stroke or intracranial ischemia or cerebral arterial thrombosis).mp. [mp=title, abstract, heading word, drug trade name, original title, device manufacturer, drug manufacturer, device trade name, keyword heading word, floating subheading word, candidate term word] 208467  #5: exp *brain ischemia/ or exp *ischemic stroke/ 89426  #6: exp *brain infarction/ 21388  #7: 4 or 5 or 6 225740  #8: 3 and 7 1230 |
| --- |

**Table 2: Basic information of included studies**

| **NO.** | **Author (year)** | **Country** | **Type of study** | **Characteristics of animals** | | | | **Sample size (E/C)** | **Modeling method** | **Characteristics of NSCs** | | **Transplantation way** | **Transplanting time (After modeling/days)** | **Intervention** | |
| --- | --- | --- | --- | --- | --- | --- | --- | --- | --- | --- | --- | --- | --- | --- | --- |
|  |  |  |  | **Species** | **Gender** | **Weight** | **Age** |  |  | **Type** | **Sources** |  |  | **Experience** | **Control** |
| 1 | Abeysinghe 2015 | Australia | RCT | Wistar rats | Male | 300-360g | Adult | 7/7 | Focal cerebral ischemia was induced i by constriction of the right MCA with perivascular administration of endothelin-1. | NSCs | Subventricular zone of human fetal brain tissues | Striatum and cortex | 7 | 8×10^5^ | Vehicle |
| 2 | Chen 2009 | China | RCT | Wistar rats | Both male and female | 250-300g | Adult | 5/5 | A nylon monofilament was inserted from the lumen of the ECA to the lumen of ICA to occlude the origin of the right middle cerebral artery for two hours. | NSCs (GDNF) | The cerebral cortex of Wistar rats | Ventricle | 3 | 5×10^5^ | Normal saline |
| 3 | Shen 2010 | China | RCT | Wistar rats | Male | / | Adult | 12/12 | A 3-0 nylon monofilament suture was gently inserted (approximately 18 mm) into the internal carotid artery. After 60 min of MCAO, the nylon surgical thread was removed. | NSCs | Wistar rat fetal forebrain | Femoral vein | 0 |  | Vehicle |
| 4 | Cheng 2015 | China | RCT | SD rats | Male | 270-300g | Adult | 18/18 | Transient focal cerebral ischemia was induced using the intraluminal thread occlusion of middle cerebral artery (MCA) for 120 min | NSCs | The neonatal mouse cerebellum | Caudal vein | 1 | 5×10^6^ | PBS |
| 5 | Doeppner 2014 | India | RCT | C57BL6/J mice | Male | 23-25g | 10-12 weeks | 6/6 | Mice were subjected to middle cerebral artery occlusion (MCAO) for 30 min under 1% isoflurane plus 69% N2O and 30% O2 anesthesia | NSCs | C57BL6/J mice | Caudal vein | 0 | 1×10^6^ | PBS |
| 6 | Gomi 2012 | Japan | RCT | C57BL6/J mice | Male | 20-25g | 6-8 weeks | 8/8 | A silicon-coated No. 8-0 nylon monofilament was placed into the internal carotid artery to confirm rCBF dropping to 80% of its former value for 30min. | NSCs | Human iPS cells | Striatum | 7 |  | Medium |
| 7 | Hicks 2008 | Canada | RCT | SD rats | Male | 320-350g | 12 weeks | 6/6 | The vasoconstrictive peptide endothelin-1 was injected adjacent to the right middle cerebral artery over 10 min as routinely done. | NSCs | GFP mice | Sensory motor cortex and Striatum | 7 | 1×10^6^ | DMEM |
| 8 | Hosseini 2015 | Iran | RCT | SD rats | Male | 200-250g | / | 10/10 | The flow was coated with 4.0 nylon head thread with silicone and inserted into the total carotid artery to block blood flow for 60 min | NSCs | Ganglion of SD rat embryo | Ventricle | 7 | 1×10^5^ | PBS |
| 9 | Hou 2016 | China | RCT | Kunming mice | Both male and female | 22-25g | 12 weeks | 10/10 | After drilling the skull to 50-μm in thickness, Rose Bengal dye pre-injected to the mice was illuminated by epifluorescence microscope to introduce photothrombotic ischemia. | NSCs | The hippocampus, olfactory bulb, and cerebral cortex of newborn Kunming mice | Cerebral cortex | 0 | 2.5×10^5^ | PBS |
| 10 | Huang 2014 | USA | RCT | C57BL6/J mice | Male | 24-26g | / | 14/14 | A 6-0 nylon monofilament coated with silicon rubber was introduced into the left internal carotid artery occlude the origin of the MCA for 60min. | NSCs | The intraventricular area of the dead fetus | Hippocampus | 1 | 1×10^5^ | PBS |
| 11 | Ishibashi 2004 | Japan | RCT | Mongolian gerbils | Male | 60-72g | 16-22 weeks | 7/7 | The left common carotid artery was occluded with a mini vascular clip for 10 min | NSCs | Forebrain tissues of human fetus | Caudate nucleus | 4 | 5×10^5^ | Medium |
| 12 | Jin 2010 | USA | RCT | Fisher 344 rats | Male | / | 3 months | 6/6 | The left MCA was occluded by electrocoagulation without damaging the brain surface | NSCs | hESC line BG01 | Infarcted area | 21 | 1.8×10^6^ | Vehicle |
| 13 | Kim 2014 | Korea | RCT | SD rats | Male | 250-300g | 2 months | 10/10 | A nylon thread was inserted into the left internal carotid artery and advanced to the Circle of Willis (permanent middle cerebral artery occlusion: pMCAo). | NSCs (PSA-NCAM+) | embryoid bodies (EBs) derived from hESCs | Striatum | 2 | 5×10^5^ | PBS |
| 14 | Song 2015 | Korea | RCT | SD rats | Male | 250-300g | / | 9/9 | Transient focal cerebral ischemia was induced using intraluminal thread occlusion of the left middle cerebral artery (MCAO) | NSCs | Fetal human brain | Caudal vein | 1 | 4×10^6^ | PBS |
| 15 | Ryu 2016 | Korea | RCT | SD rats | Male | 270-300g | / | 7/7 | By intraluminal thread occlusion of the left middle cerebral artery (MCAo) for 2 hours followed by reperfusion | NSCs | Immortalized human neural stem cell | Ventricle | 1 | 6×10^5^ | PBS |
| 16 | Kim 2019 | Korea | RCT | SD rats | Male | 260-280g | 7 weeks | 10/10 | The middle cerebral artery was blocked with silicone thread for 2 hours | NSCs (over-expressing choline acetyltransferase) | Human | Caudal vein | / | 1×10^6^ | Normal saline |
| 17 | Kim 2020 | Korea | RCT | ICR mice | / | / | 1 week | 10/10 | ICR mice were subjected to permanent right common carotid artery occlusion under isoflurane anesthesia | NSCs | Human fetal brain tissue | Infarcted area | 3 | 8×10^5^ | Vehicle |
| 18 | Lacza 2003 | USA | RCT | Wistar rats | Male | / | Adult | 12/11 | A nickel coated brass stamp with a footprint of 3 mm in diameter was precooled to -60°C with a mixture of dry ice and acetone and was lowered by a micromanipulator to touch the exposed dura for 3 min | NSCs (PARP) | E14 rat embryos | Infarcted area | 6 | 1.5×10^5^ | PBS |
| 19 | Chang 2013 | Korea | RCT | SD rats | Male | 270-300g | Adult | 8/6 | A blunt-ended monofilament was inserted into the internal carotid artery from the bifurcation to occlude the middle cerebral artery for 90min. | NSCs | H9 hESCs | Striatum | 7 | 2×10^5^ | Medium |
| 20 | Lei 2012 | China | RCT | SD rats | Male | 200-240g | / | 18/6 | The middle cerebral artery was isolated, occluded with a nylon monofilament suture with a rounded tip using an intraluminal vascular occlusion method. | NSCs | Cortex and ganglionic eminence of SD fetal rats | Ventricle | 3 | 2×10^5^ | PBS |
| 21 | Zhang 2009 | China | RCT | SD rats | Male | 250-300g | / | 30/30 | A 3-0 surgical monofilament nylon suture with a rounded tip was introduced into the left internal carotid through the arteriotomy and advanced 16.5–17.5mm past the carotid bifurcation. | NSCs | Embryonic human brains | Cerebral cortex | 1 | 5×10^4^ | Medium |
| 22 | Li 2013 | China | RCT | SD rats | Male | 300g | Adult | 6/6 | / | NSCs | Forebrain tissue of embryonic SD rats | Cerebral cortex | 3 | 5×10^5^ | PBS |
| 23 | Lu 2017 | China | RCT | ICR mice | Male | / | Adult | 7/7 | The middle cerebral artery (MCA) was occluded by a 6–0 suture with a silicone-coated round tip. | NSCs | Cortex of E14 transgenic mice | Striatum | 4 | 3×10^5^ | PBS |
| 24 | Zhu 2017 | China | RCT | Wistar rats | Male | 250-280g | 12-16weeks | 20/20 | Closure of middle cerebral artery with nylon suture for 120 minutes | NSCs | cortex of newborn Wistar rats | Striatum | 3 | 5×10^5^ | PBS |
| 25 | Ma 2015 | China | RCT | C57BL6/J mice | Male | 20-25g | Adult | 6/6 | The cortical micro vessels of the thinned region were illuminated with a beam of green light for 3 min. | NSCs | Hippocampus tissue of mice | Hippocampus | 2 | 1×10^6^ | PBS |
| 26 | Mochizuki 2008 | Japna | RCT | Wistar rats | Male | 220-260g | / | 7/7 | / | NSCs | Brain of fetal rats | Cerebral cortex | 0 | 1×10^5^ | Vehicle |
| 27 | MOCHIZUKI 2011 | Japna | RCT | Wistar rats | Male | 220-260g | / | 8/8 | / | NSCs | Brain of fetal rats | Hippocampus | 7 | 1×10^5^ | Vehicle |
| 28 | Noh 2020 | Korea | RCT | SD rats | Male | 270-300g | Adult | 10/10 | A blunt-ended silicon-coated monofilament was inserted to occlude the middle cerebral artery (MCA) for 90 minutes before it was removed. | NSCs | Human-induced pluripotent stem cells | Striatum | 7 | 2×10^5^ | Medium |
| 29 | Zhu 2004 | China | RCT | SD rats | Male | 250-300g | Adult | 10/10 | 120 min occlusion of middle cerebral artery by suture | NSCs | hippocampus of Day E14 fetal SD rats | Infarcted area | 3 | 2×10^5^ | PBS |
| 30 | Rehni 2007 | China | RCT | Albino mice | Both male and female | 25±2g | / | 6/6 | The suture was allowed to remain at the origin of middle cerebral artery to produce partial occlusion of cerebral artery for 1h. | NSCs | Brain tissue of the pregnant mouse fetus | Ventricle | 3 | 5×10^5^; 1×10^6^ | PBS |
| 31 | Saraiva 2018 | Portuga | RCT | C57BL6/J mice | Male | / | 9 weeks | 11/16 | Unilateral photothrombotic cortical ischemia in the right primary motor cortex | NSCs | C57BL6/J mice | Caudal vein | 0 |  | Normal saline |
| 32 | Song 2010 | Korea | RCT | SD rats | Male | 250-300g | / | 12/10 | Transient focal cerebral ischemia was induced by intralumenal thread occlusion of the left middle cerebral artery. | NSCs | Fetal human brain cell | Caudal vein | 1 | 4×10^6^ | PBS |
| 33 | Song 2011 | Korea | RCT | SD rats | Male | 250-300g | / | 6/6 | Transient focal cerebral ischemia was induced by intralumenal thread occlusion of the left middle cerebral artery. | NSCs | human neural stem cell | Caudal vein | 1/7 | 4×10^6^ | PBS |
| 34 | Song 2015 | Korea | RCT | SD rats | Male | 250-300g | / | 12/10 | Transient focal cerebral ischemia was induced by intralumenal thread occlusion of the left middle cerebral artery. | NSCs | human neural stem cell | Caudal vein | 1 | 4×10^6^ | PBS |
| 35 | Ryu 2015 | Korea | RCT | SD rats | Male | 270-300g | / | 7/7 | By intraluminal thread occlusion of the left middle cerebral artery (MCAo) for 2 hours followed by reperfusion | NSCs | Human | Ventricle | 1 | 6×10^5^ | PBS |
| 36 | Takahashi 2008 | Japan | RCT | Wistar rats | Male | 250-280g | Adult | 8/8 | A 4-0 monofilament nylon suture with the tip polysiloxane-coated was advanced from the CCA bifurcation until it blocked the origin of the MCA for 90min | NSCs | Neural crest of Wistar rat embryos | Infarcted area | 1h | 1×10^5^ | Vehicle |
| 37 | Tang 2014 | China | RCT | SD rats | Male | / | 24 months | 17/16 | A 4-0 round tip and silicon coated suture was inserted from the left external carotid artery into the internal carotid artery for 2h. | NSCs | GFP mice | Striatum | 1 | 1×10^6^ | PBS |
| 38 | Ziaee 2017 | Iran | RCT | SD rats | Male | 250-300g | 10-12 weeks | 15/15 | 30 min occlusion of middle cerebral artery with nylon thread | NSCs | embryo rat ganglion eminence | Ventricle | 1/3/7 | 2×10^5^ | PBS |
| 39 | Tian 2019 | China | RCT | SD rats | Male | 280-300g | / | 10/10 | The rats were subjected to transient (2 h) MCAo via right intraluminal vascular occlusion | NSCs (Leukemia inhibitory factor) | The cortex of embryonic mouse brains | Caudal vein | 6h | 5×10^6^ | Blank |
| 40 | Wang 2019 | China | RCT | SD rats | Male | / | / | 12/12 | The 0.25 mm nylon suture was inserted into the arterial origin through ICA until resistance was encountered. Fixed nylon suture, suture incision. | NSCs | Fetuses were harvested from 15-d-pregnant Sprague-Dawley rat | Striatum | 1 | 1×10^6^ | Blank |
| 41 | Watanabe 2015 | Japan | RCT | Wistar rats | Male | 250-300g | Adult | 5/5 | 4 – 0 monofilament nylon sutures (20 mm) were used to block the origin of middle cerebral artery for 60 min. | NSCs (BRCA1) | human fetal telencephalonn | Jugular vein | 6h | 3×10^6^ | PBS |
| 42 | Xu 2019 | China | RCT | C57BL6/J mice | Male | 20-25g | Adult | 8/8 | A 6-0 siliconcoated monofilament nylon suture was introduced into the ICA through the ECA until mild resistance was felt for 90min. | NSCs | embryonic C57BL/6J mouse brain | Cerebral cortex | 7h | 1×10^6^ | PBS |
| 43 | Yamashita 2017 | Japan | RCT | C57BL6/J mice | Male | 23-27g | 8-10 weeks | 19/19 | A silicone-coated 6-0 filament was inserted and gently advanced (9.0–10.0 mm) to occlude the middle cerebral artery for 35min. | NSCs | C57BL6/J mice | Striatum and cortex | 1 | 5×10^5^ | Normal saline |
| 44 | Yao 2015 | China | RCT | SD rats | Male | 270-300g | / | 12/12 | / | NSCs (human neurotrophin-3) | embryos of C57BL6/J mice | Cerebral cortex | 2 | 1×10^6^ | PBS |
| 45 | Zhang 2008 | China | RCT | SD rats | Male | 230-250g | Adult | 12/12 | A 3–0 monofilament nylon suture with a round tip was introduced into the CCA lumen and gently advanced to the ICA until slight resistance was left for 120min. | NSCs | Embryonic hippocampus of SD rats | Striatum | 7 | 2×10^5^ | Normal saline |
| 46 | Zhang 2017 | China | RCT | SD rats | Male | 270-300g | Adult | 16/16 | A 5-0 monofilament nylon suture was advanced from the ECA into the lumen of the ICA until a slight resistance was exerted (18.5–19.5 mm) for 120min. | NSCs (bFGF) | mice | Caudal vein | 1 | 5×10^6^ | PBS |
| 47 | Zhang 2018 | China | RCT | SD rats | Male | 240-280g | Adult | 15/15 | Nylon thread was introduced into the CCA. The insertion depth was approximately 18 mm ± 0.5 mm above the CCA bifurcation for 90min. | NSCs | hippocampus of fetal rats | Striatum | 3 | 8×10^5^ | PBS |
| 48 | Zhang 2017 | China | RCT | SD rats | Male | 250-280g | Adult | 6/6 | The insertion length of the sutures was 2 cm. After 120 min of occlusion, the suture was withdrawn to restore blood supply to the MCA territory | NSCs | newborn SD rat | Striatum | 1 | 5×10^5^ | PBS |
| 49 | Zhang 2017 | China | RCT | ICR mice | Male | 25-30g | Adult | 8/8 | The middle cerebral arteries were closed with a suture for 2 hours | NSCs | The cortex of GFP mice | Striatum | 1 | 1×10^6^ | PBS |
| 50 | Zhang 2018 | China | RCT | SD rats | Male | 250-300g | Adult | 24/24 | Closure of middle cerebral artery with nylon suture for 90 minutes | NSCs | hippocampus of fetal rats | Striatum | 0 | 8×10^5^ | PBS |
| 51 | Zhu 2005 | China | RCT | SD rats | Male | 250-300g | Adult | 10/10 | Closure of middle cerebral artery with nylon suture for 120 minutes | NSCs | hippocampus of E14 fetal SD rats | Striatum | 3 | 2×10^5^ | PBS |
| 52 | Zhu 2011 | China | RCT | SD rats | Male | 250-300g | Adult | 10/10 | Closure of middle cerebral artery with nylon suture for 120 minutes | NSCs | hippocampus of E14 fetal SD rats | Striatum | 3 | 1×10^6^ | PBS |

**Table 3: Results of network meta-analysis among neural stem cells with different transplantation doses in rats**

| 1×10^5^ | 27.83 (-24.37, 80.61) | 28.92 (-46.43, 104.60) | -3.19 (-64.82, 57.54) | 21.82 (-37.12, 82.41) | 40.99 (-32.40, 116.86) | 35.71 (-26.70, 96.56) | 37.14 (-17.83, 93.99) | 24.94 (-35.82, 86.18) | 36.58 (-23.69, 97.17) | 43.21 (0.23, 86.34) |
| --- | --- | --- | --- | --- | --- | --- | --- | --- | --- | --- |
| -27.83 (-80.61, 24.37) | 1×10^6^ | 1.25 (-68.60, 70.63) | -31.00 (-86.06, 22.84) | -6.47 (-56.60, 46.82) | 13.23 (-54.67, 80.60) | 7.64 (-45.99, 60.07) | 9.67 (-36.61, 56.36) | -2.95 (-54.93, 51.01) | 9.48 (-44.13, 61.35) | 15.29 (-15.23, 45.72) |
| -28.92 (-104.60, 46.43) | -1.25 (-70.63, 68.60) | 1.8×10^6^ | -32.11 (-108.81, 41.74) | -7.34 (-81.30, 69.23) | 12.31 (-75.34, 100.51) | 7.09 (-68.88, 81.60) | 8.82 (-62.71, 78.88) | -3.95 (-78.85, 71.04) | 8.17 (-67.98, 84.21) | 14.13 (-48.52, 76.49) |
| 3.19 (-57.54, 64.82) | 31.00 (-22.84, 86.06) | 32.11 (-41.74, 108.81) | 2×10^5^ | 24.89 (-34.20, 87.43) | 44.42 (-29.94, 121.40) | 38.59 (-23.21, 100.40) | 40.83 (-14.99, 96.46) | 27.95 (-32.46, 90.09) | 40.25 (-21.86, 103.15) | 46.40 (2.15, 91.35) |
| -21.82 (-82.41, 37.12) | 6.47 (-46.82, 56.60) | 7.34 (-69.23, 81.30) | -24.89 (-87.43, 34.20) | 4×10^6^ | 19.44 (-55.95, 92.55) | 14.05 (-48.68, 74.20) | 15.70 (-40.36, 70.22) | 3.32 (-56.30, 62.41) | 15.01 (-46.34, 74.82) | 21.43 (-21.88, 62.37) |
| -40.99 (-116.86, 32.40) | -13.23 (-80.60, 54.67) | -12.31 (-100.51, 75.34) | -44.42 (-121.40, 29.94) | -19.44 (-92.55, 55.95) | 5×10^4^ | -5.70 (-82.68, 70.30) | -3.54 (-74.09, 65.43) | -16.43 (-90.24, 58.59) | -4.28 (-79.42, 70.07) | 2.18 (-59.64, 63.00) |
| -35.71 (-96.56, 26.70) | -7.64 (-60.07, 45.99) | -7.09 (-81.60, 68.88) | -38.59 (-100.40, 23.21) | -14.05 (-74.20, 48.68) | 5.70 (-70.30, 82.68) | 5×10^5^ | 1.72 (-54.06, 59.38) | -10.57 (-70.98, 50.82) | 1.30 (-58.74, 63.18) | 7.66 (-35.23, 51.87) |
| -37.14 (-93.99, 17.83) | -9.67 (-56.36, 36.61) | -8.82 (-78.88, 62.71) | -40.83 (-96.46, 14.99) | -15.70 (-70.22, 40.36) | 3.54 (-65.43, 74.09) | -1.72 (-59.38, 54.06) | 5×10^6^ | -12.39 (-67.90, 43.54) | -0.00 (-56.62, 54.15) | 5.60 (-28.83, 40.37) |
| -24.94 (-86.18, 35.82) | 2.95 (-51.01, 54.93) | 3.95 (-71.04, 78.85) | -27.95 (-90.09, 32.46) | -3.32 (-62.41, 56.30) | 16.43 (-58.59, 90.24) | 10.57 (-50.82, 70.98) | 12.39 (-43.54, 67.90) | 6×10^5^ | 11.77 (-49.78, 72.66) | 18.03 (-26.33, 60.81) |
| -36.58 (-97.17, 23.69) | -9.48 (-61.35, 44.13) | -8.17 (-84.21, 67.98) | -40.25 (-103.15, 21.86) | -15.01 (-74.82, 46.34) | 4.28 (-70.07, 79.42) | -1.30 (-63.18, 58.74) | 0.00 (-54.15, 56.62) | -11.77 (-72.66, 49.78) | 8×10^5^ | 6.36 (-35.66, 49.76) |
| -43.21 (-86.34, -0.23) | -15.29 (-45.72, 15.23) | -14.13 (-76.49, 48.52) | -46.40 (-91.35, -2.15) | -21.43 (-62.37, 21.88) | -2.18 (-63.00, 59.64) | -7.66 (-51.87, 35.23) | -5.60 (-40.37, 28.83) | -18.03 (-60.81, 26.33) | -6.36 (-49.76, 35.66) | Placebo |

**Table 4: Results of network meta-analysis among neural stem cells with different transplantation doses in mice**

| 1×10^5^ | 13.67 (-2.19, 20.75) | 6.21 (-0.69, 8.52) | 11.55 (-5.82, 12.04) | 2.72 (-0.07, 10.58) | 9.54 (-1.96, 15.17) | 13.13 (-8.91, 14.36) |
| --- | --- | --- | --- | --- | --- | --- |
| -13.67 (-20.75, 2.19) | 1×10^6^ | -8.49 (-14.22, 1.17) | -2.54 (-8.92, 4.68) | -7.80 (-16.46, 2.12) | -1.93 (-18.03, 7.82) | -0.20 (-7.38, 7.01) |
| -6.21 (-8.52, 0.69) | 8.49 (-1.17, 14.22) | 2.5×10^5^ | 5.34 (-3.51, 6.51) | -0.80 (-6.59, 4.05) | 6.25 (-3.93, 7.38) | 6.92 (-5.84, 9.60) |
| -11.55 (-12.04, 5.82) | 2.54 (-4.68, 8.92) | -5.34 (-6.51, 3.51) | 3×10^5^ | -6.65 (-10.11, 1.46) | 0.61 (-9.11, 3.14) | 2.07 (-1.33, 3.09) |
| -2.72 (-10.58, 0.07) | 7.80 (-2.12, 16.46) | 0.80 (-4.05, 6.59) | 6.65 (-1.46, 10.11) | 5×10^5^ | 4.21 (-1.56, 13.25) | 9.10 (-2.79, 12.44) |
| -9.54 (-15.17, 1.96) | 1.93 (-7.82, 18.03) | -6.25 (-7.38, 3.93) | -0.61 (-3.14, 9.11) | -4.21 (-13.25, 1.56) | 8×10^5^ | 1.60 (-0.81, 10.92) |
| -13.13 (-14.36, 8.91) | 0.20 (-7.01, 7.38) | -6.92 (-9.60, 5.84) | -2.07 (-3.09, 1.33) | -9.10 (-12.44, 2.79) | -1.60 (-10.92, 0.81) | Placebo |

**Table 5: Ranking results between different doses of neural stem cells transplantation in rats**

| Drug | Rank 1 | Rank 2 | Rank 3 | Rank 4 | Rank 5 | Rank 6 | Rank 7 | Rank 8 | Rank 9 | Rank 10 | Rank 11 |
| --- | --- | --- | --- | --- | --- | --- | --- | --- | --- | --- | --- |
| 1×10^5^ | 0.03 | 0.01 | 0.01 | 0.01 | 0.03 | 0.04 | 0.06 | 0.09 | 0.16 | 0.27 | 0.32 |
| 1×10^6^ | 0.00 | 0.05 | 0.07 | 0.10 | 0.14 | 0.15 | 0.17 | 0.14 | 0.10 | 0.05 | 0.01 |
| 1.8×10^6^ | 0.00 | 0.10 | 0.07 | 0.06 | 0.08 | 0.09 | 0.09 | 0.10 | 0.11 | 0.09 | 0.08 |
| 2×10^5^ | 0.14 | 0.01 | 0.01 | 0.01 | 0.02 | 0.03 | 0.05 | 0.08 | 0.14 | 0.25 | 0.41 |
| 4×10^6^ | 0.04 | 0.04 | 0.06 | 0.07 | 0.09 | 0.10 | 0.13 | 0.14 | 0.15 | 0.11 | 0.06 |
| 5×10^4^ | 0.28 | 0.13 | 0.08 | 0.07 | 0.08 | 0.07 | 0.07 | 0.07 | 0.06 | 0.05 | 0.04 |
| 5×10^5^ | 0.13 | 0.12 | 0.11 | 0.11 | 0.11 | 0.11 | 0.10 | 0.09 | 0.06 | 0.04 | 0.02 |
| 5×10^6^ | 0.12 | 0.14 | 0.13 | 0.13 | 0.13 | 0.13 | 0.10 | 0.07 | 0.04 | 0.02 | 0.01 |
| 6×10^5^ | 0.05 | 0.06 | 0.07 | 0.09 | 0.09 | 0.12 | 0.12 | 0.13 | 0.13 | 0.09 | 0.05 |
| 8×10^5^ | 0.15 | 0.14 | 0.11 | 0.10 | 0.10 | 0.11 | 0.10 | 0.08 | 0.06 | 0.03 | 0.01 |
| Placebo | 0.06 | 0.21 | 0.29 | 0.25 | 0.13 | 0.05 | 0.01 | 0.00 | 0.00 | 0.00 | 0.00 |

**Table 6: Ranking results between different doses of neural stem cells transplantation in mice**

| Drug | Rank 1 | Rank 2 | Rank 3 | Rank 4 | Rank 5 | Rank 6 | Rank 7 |
| --- | --- | --- | --- | --- | --- | --- | --- |
| 1×10^5^ | 0.00 | 0.00 | 0.00 | 0.00 | 0.00 | 0.25 | 0.75 |
| 1×10^6^ | 0.50 | 0.00 | 0.00 | 0.25 | 0.25 | 0.00 | 0.00 |
| 2.5×10^5^ | 0.00 | 0.00 | 0.00 | 0.50 | 0.00 | 0.25 | 0.25 |
| 3×10^5^ | 0.00 | 0.00 | 0.75 | 0.25 | 0.00 | 0.00 | 0.00 |
| 5×10^5^ | 0.00 | 0.00 | 0.00 | 0.00 | 0.75 | 0.25 | 0.00 |
| 8×10^5^ | 0.25 | 0.25 | 0.25 | 0.00 | 0.00 | 0.25 | 0.00 |
| Placebo | 0.25 | 0.75 | 0.00 | 0.00 | 0.00 | 0.00 | 0.00 |


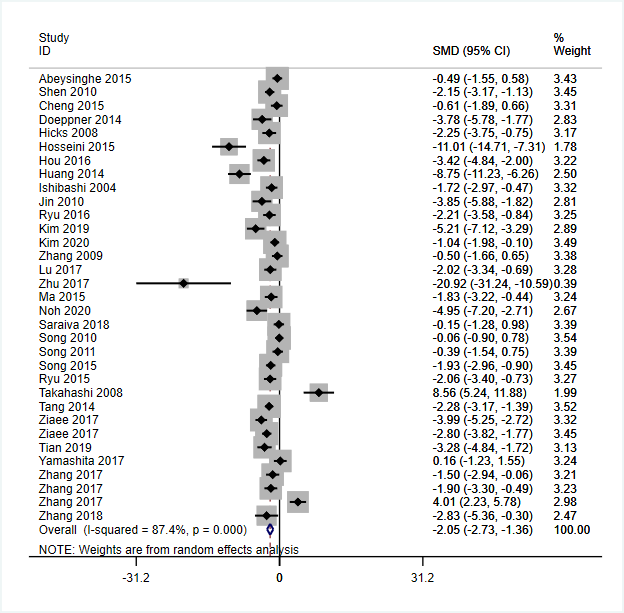


**Figure 1: Traditional meta-analysis results of cerebral infarction volume**


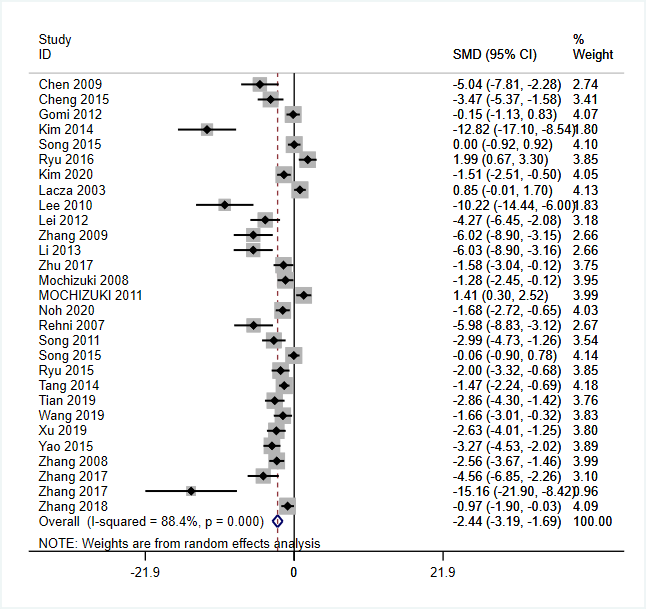


**Figure 2: Traditional meta-analysis results of the first week of mNSS**


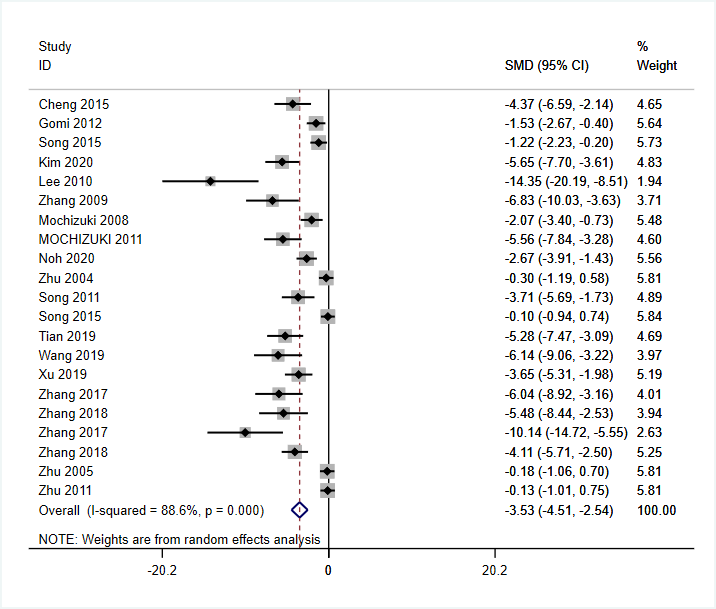


**Figure 3: Traditional meta-analysis results of the fourth week of mNSS**
